# Genome-Wide Identification and Characterization of Heat Shock Protein 20 Genes in Maize

**DOI:** 10.3390/life12091397

**Published:** 2022-09-08

**Authors:** Huanhuan Qi, Xiaoke Chen, Sen Luo, Hongzeng Fan, Jinghua Guo, Xuehai Zhang, Yinggen Ke, Pingfang Yang, Feng Yu

**Affiliations:** 1 State Key Laboratory of Biocatalysis and Enzyme Engineering, School of Life Sciences, Hubei University, Wuhan 430062, China; 2 National Key Laboratory of Wheat and Maize Crop Science, Henan Agricultural University, Zhengzhou 450002, China

**Keywords:** heat shock protein 20, maize, abiotic stress, yeast-one-hybrid

## Abstract

Maize is an important cereal crop worldwide and is sensitive to abiotic stresses in fluctuant environments that seriously affect its growth, yield, and quality. The small heat shock protein (*HSP20*) plays a crucial role in protecting plants from abiotic stress. However, little is known about *HSP20* in maize (*ZmHSP20*). In this study, 44 *ZmHSP20s* were identified, which were unequally distributed over 10 chromosomes, and 6 pairs of *ZmHSP20s* were tandemly presented. The gene structure of *ZmHSP20s* was highly conserved, with 95% (42) of the genes having no more than one intron. The analysis of the cis-element in *ZmHSP20s* promoter demonstrated large amounts of elements related to hormonal and abiotic stress responses, including abscisic acid (ABA), high temperature, and hypoxia. The ZmHSP20s protein had more than two conserved motifs that were predictably localized in the cytoplasm, nucleus, endoplasmic reticulum, peroxisome, mitochondria, and plasma. Phylogenetic analysis using HSP20s in *Arabidopsis*, rice, maize, and *Solanum tuberosum* indicated that ZmHSP20s were classified into 11 categories, of which each category had unique subcellular localization. Approximately 80% (35) of *ZmHSP20* were upregulated under heat stress at the maize seedling stage, whereas the opposite expression profiling of 10 genes under 37 and 48 °C was detected. A total of 20 genes were randomly selected to investigate their expression under treatments of ABA, gibberellin (GA), ethylene, low temperature, drought, and waterlogging, and the results displayed that more than half of these genes were downregulated while *ZmHSP20-3*, *ZmHSP20-7*, *ZmHSP20-24*, and *ZmHSP20-44* were upregulated under 1 h treatment of ethylene. A yeast-one-hybrid experiment was conducted to analyze the binding of four heat stress transcription factors (ZmHSFs) with eight of the *ZmHSP20s* promoter sequences, in which ZmHSF3, ZmHSF13, and ZmHSF17 can bind to most of these selected *ZmHSP20s* promoters. Our results provided a valuable resource for studying *HSP20s* function and offering candidates for genetic improvement under abiotic stress.

## 1. Introduction

In the changing environment, numerous adverse stress conditions such as drought, salinity, heat, cold, and chemicals, nematodes, insects, and rodents were imposed on plants, which significantly influence their growth and development [1]. These abiotic stresses can cause damage to plant cells and cause secondary damage, such as osmotic and oxidative stress [2,3]. Plants have a series of elaborate mechanisms in response to environmental changes compared to animals, including maintaining cell membrane stability [4], capturing reactive oxygen species (ROS), synthesizing antioxidants, osmotic accumulation, and osmotic regulation, inducing some enzymes in response to stress, and enhancing the transcription and signaling of partners [5], to adapt morphologically and physiologically [6]. Abiotic stresses in plants are often interrelated and lead to physiological, morphological, cellular, and molecular changes [1], and two or more abiotic stresses are often more lethal than single stress [7]. 

Heat shock protein (*HSP*) in *Drosophila melanogaster* was primarily discovered under exposure to high-temperature stress [8]. The response to stresses on the molecular level was found in all organisms, especially the sudden changes in genotypic expression resulting in an increase in the synthesis of *HSP* proteins [9,10,11]. The *HSPs* are characterized by the presence of a carboxyl terminus called a heat shock domain [12]. Under environmental stress conditions, plants reduce the synthesis of normal proteins and facilitate the transcription and translation of *HSPs* [13]. The expression of *HSPs* is mediated by the binding of heat stress transcription factors (HSFs) to heat shock element (HSE) sequences that are located in the promoter region of *HSPs* [2]. The heat shock promoter is characterized by a conserved palindromic element with the consensus motif “nGAAnnTTCn”. This HSE motif or its various variants have different effects on the interaction of HSFs with HSE [14]. *HSPs* can be divided into five classes according to their molecular weight and sequence homology, including *HSP100*, *HSP90*, *HSP70*, *HSP60*, and Small *HSPs* (*HSP20*) [15,16], in which *HSP20s* are 12–25 kDa polypeptides. Most *HSP20s* occur together in oligomers with 12 subunits. Plants have many types of *HSP20s*, and some species have more than 40 types of *HSP20s* [15]. The structure of *HSP20s* presents remarkable diversity, but all *HSP20s* share a common α-crystalline domain (ACD) that allows them to be recognized [17], reflecting their fitness with diversity stresses. *HSP20s* are widely present in plants and help to protect plant cells against protein breakdown and maintain their functional conformation [18]. *HSP20s* can also act as an ATP-independent molecular chaperone to capture substrate proteins denatured by stress [19], preventing the irreversible denaturation of substrates [20]. The feature facilitates the refolding of denatured proteins and improves plant performance in adapting to environmental stress. 

Most *HSP20s* are not expressed under normal conditions but can be omnipresent in various biotic and abiotic stresses [21]. *HSP20s* are thus considered a component of cell protein quality control to defend against stresses and coordinate defensive signaling cascades by participating in the build-up of various resistance proteins. *HSP20s* are also involved in plant embryogenesis, germination, and fruit development. For example, *HSP21* in tomato participates in the accumulation of carotenoids during ripening [22]. *HSP20* plays an important role in abiotic stress and has been identified in various plants. *PtHSP17.8* of *Populus trichocarpa* enhances heat and salt tolerance by maintaining ROS homeostasis and collaboration [23]. Overexpression of maize *HSP16.9* in tobacco can increase heat tolerance and oxidation resistance [24]. The expression of *HSP22.8* in watermelon is reduced under abscisic acid (ABA) stress and salt stress [25]. *HSP17.7* in tomato can maintain intracellular Ca^2+^ homeostasis and improve cold tolerance [26]. Moreover, most *HSP20s* in apple were upregulated under heat stress [27].

Maize (*Zea mays*) is an essential staple crop in Latin America, Asia, and sub-Saharan Africa, mainly for human consumption and animal feed production [28]. Aside from its agronomic importance, maize has been a key model for fundamental research for almost a century [29]. However, the *HSP20* gene family in maize (*ZmHSP20s*) has not been fully researched [30,31]. In this study, we systematically identified and characterized *ZmHSP20s* in maize genome, which included the gene structure, conserved motif, cis-element in the promoter, and phylogenetic relationship. We also analyzed the expression level of *ZmHSP20s* under hormone treatments and abiotic stresses, especially for expression levels under high temperatures. Moreover, the possible interactions between ZmHSP20s and ZmHSFs were experimentally verified. These results provide valuable resources for investigating the function of *HSP20s* in plants.

## 2. Materials and Methods

### 2.1. Plant Growth and Treatment

Seeds of the maize inbred line B73 were planted in a greenhouse with a controlled temperature (~25 °C/22 °C, day/light cycle), a 14 h/10 h light/dark cycle, and 60% average humidity. As previously described, the treatments were imposed on seedlings at the second leaf stage [32]. For the high-temperature treatment, the seedlings were transferred to an artificial climate chamber at 37, 42, and 48 °C, and the leaves were collected after 4 h of stress. The seedling leaves under 10 °C, drought stress after 1, 2, and 4 h treatments, and waterlogged roots were also collected. For hormone treatments, 100 μM of ethylene (ET), 100 mM of ABA, and 100 mM of gibberellin (GA) were applied to treat the seedlings, and the leaves after 1, 2, and 4 h treatments were sampled. The leaves and roots of seedlings growing under 25 °C conditions were collected as the control. For each sample, more than six seedlings were mixed and immediately frozen at −80 °C.

### 2.2. RNA Extraction and Quantitative Reverse Transcription PCR (qRT-PCR)

Total RNA was isolated using TRIZOL reagent (Invitrogen, Gaithersburg, MD, USA) and treated with RNase-free DNase (Invitrogen). Purified RNA was used to synthesize single-stranded cDNA using recombinant M-MLV reverse transcriptase (Invitrogen). Quantitative reverse transcription PCR (qRT-PCR) was performed using gene-specific primers (Table S1) and a 2 × iTaq^TM^ Universal SYBR Green Super Mix (Bio-Rad, Hercules, CA, USA). *ZmActin1* (*GRMZM2G126010*) was used as an internal control for the normalization of expression data. Relative expression levels were calculated using the 2^−ΔΔCT^ (cycle threshold) method [33]. PCR involved an initial denaturation step at 95 °C for 5 min, followed by 40 cycles of 15 s at 95 °C, 10 s at 58 °C, and 20 s at 72 °C. The primers used for qRT-PCR were designed using online software Primer3Plus (https://www.primer3plus.com/ (accessed on 1 June 2022)).

### 2.3. Identification of ZmHSP20s

Two approaches were applied to identify the *ZmHSP20s* family genes in maize. The conserved *ZmHSP20* domain (PF00011) from the Pfam database [34] was used to query the maize B73 proteome (RefGen_v4) [35] with the *ZmHSP20* HMM using the HMMER3.0 package [36], and the *ZmHSP20* proteins were collected based on the E-value < 1 × 10^−5^. Moreover, the protein sequences of *HSP20* family members in *Arabidopsis* and rice [37] were downloaded from TAIR [38] and the MSU Rice Genome Annotation Project [39] databases, respectively. These protein sequences from these *Arabidopsis* and rice *HSP20* were used as queries to search against the maize proteome with an E-value < 1 × 10^−5^ based on a local BLASTP program with the default parameters. The proteins from these two approaches were collected and redundant sequences were manually eliminated. The Pfam [34] and SMART [40] databases were utilized to confirm the conserved domain in the identified proteins. The molecular weight (MW) and isoelectric point (pI) of ZmHSP20s were computed with the online ExPASy tool [41]. Four online tools (Predotar [42], WOLF PSORT (https://www.genscript.com/wolf-psort.html (accessed on 6 June 2022)), TargetP [43], and CELLO [44]) were used to predict the subcellular localization. Some subcellular localizations of ZmHSP20s that cannot be predicted using software will be predicted by affinities with other species.

### 2.4. Analysis of Gene Structure, Chromosome Distribution, Duplication, Collinearity, and Conserved Motif

DNA, coding sequences (CDSs), and protein sequences of ZmHSP20 family genes and their corresponding physical location in the maize B73 reference genome (RefGen_v4) were downloaded from the MaizeGDB database. The gene structures were drawn and displayed by Gene Structure Display Server (GSDS) [45] using DNA and CDS sequences of each gene. The online program of Multiple Em for Motif Elicitation (MEME, V5.0.3, https://meme-suite.org/meme/doc/meme.html, accessed on 6 June 2022) was applied to predict the potential motifs with default parameters. The MG2C (MapGene2Chromosome V2, http://mg2c.iask.in/mg2c_v2.0/, accessed on 8 June 2022) software was used to display the physical location of each gene in its corresponding position. According to the manual, the *ZmHSP20s* gene collinearity analysis within the maize genome was conducted using MCScanX software with default parameters [46].

### 2.5. Phylogenetic Analysis

To illuminate the evolutionary relationship of ZmHSP20s, the phylogenetic tree was constructed using representative HSP20s protein sequences of *Arabidopsis thaliana* (AtHSP20s) [47], rice (OsHSP20s) [48], *Solanum tuberosum* (StHSP20s) [49], and 44 ZmHSP20s. After being aligned using ClustalW [50], the aligned sequences were imported into MEGA11 [51] to construct an unrooted neighbor-joining phylogenetic tree (NJ) using 1000 bootstrap repetitions. The phylogenetic tree was modified using the online software iTOL [52].

### 2.6. Predicting the Cis-Regulatory Elements

The 1.5 kb sequences of the promoter of *ZmHSP20* genes were obtained from the EnsemblPlants database and were then uploaded to the website of PlantCare [53] to predict the cis-regulatory DNA elements. The elements related to stress response and hormones were selected and displayed through Tbtools [54].

### 2.7. Prediction of the Interaction between ZmHSP20s and ZmHSFs

Protein sequences for *HSF* family members in maize (ZmHSFs) [55] were downloaded from maizeGDB [56], which were uploaded onto STRING database [57] to predict the interaction with ZmHSP20s. The interaction networks were drawn through Cytoscape_v3.9.1 [58]. The promoter sequences of *ZmHSP20s* were uploaded onto PlantRegMap [59] to predict the binding of ZmHSFs.

### 2.8. Yeast One- and Two-Hybrid Assays

A full-length CDS of *ZmHSFs* was cloned into vector pGADT7-Rec2 and the 1.5 kb promoter sequence of *ZmHSP20s* was cloned into vector pHIS2 using a CloneExpressII One Step Cloning Kit (Vazyme, Nanjing, China) with the corresponding primers (Table S1). Recombinant vectors were co-transfected into yeast competent *AH109*. Transformants were cultured on SD/-Leu-Trp and were then placed on SD/-Leu-Trp-His with a special concentration of 3-amino-1,2,4-triazole (3-AT). For the yeast-two-hybrid experiment, the full-length CDS of *ZmHSFs* was cloned into vector pGADT7 while the full-length CDS of *ZmHSP20s* was cloned into vector pGBKT7, and the transformants were screened on SD/-Leu-Trp-His-Ade.

## 3. Results

### 3.1. The Characters of ZmHSP20 Gene Members

**Figure 1:**
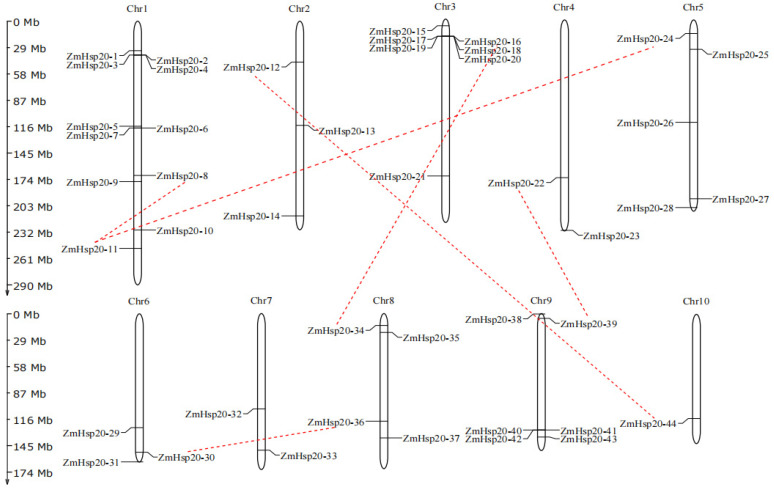
Genome-wide distribution of *ZmHSP20* genes on maize chromosomes. The chromosomal location of each *ZmHSP20* gene is annotated with the gene name. Chromosome numbers are indicated at the top of each bar. The *ZmHSP20* genes present on duplicated chromosomal segments are connected by red dashed lines.

A total of 44 members of *ZmHSP20s* in maize were finally identified through BLASP and HMMER programs, which were referred to as *ZmHSP20-1* to *ZmHSP20-44* based on their location in chromosomes (Table S2). *ZmHSP20s* locate across 10 chromosomes, and chromosomes 1 (11) and 3 (7) had the largest member of *ZmHSP20s* while chromosomes 4 (*ZmHSP20-22* and *ZmHSP20-23*), 7 (*ZmHSP20-32* and *ZmHSP20-33*), and 10 (*ZmHSP20-44*) had the smallest member of *ZmHSP20s*. The isoelectric point ranged from 4.75 (*ZmHSP20-30*) to 11.66 (*ZmHSP20-17*) and the molecular weight (MW) ranged from 13.98 to 62.73 kilodalton (Kd), most of which were around 20 Kd. *ZmHSP20-8* (62.73 Kd) and *ZmHSP20-21* (46.21 Kd) had higher apparent MWs although both proteins had a conserved domain of HSP20 (Table S2). The subcellular localization of ZmHSP20s demonstrated that most of these proteins localized in the cytoplasmic region, while some proteins localized in nuclear, mitochondrial, endoplasmic reticulum (ER), plastid, and peroxisome (Po) regions. Four gene clusters of *ZmHSP20s* in chromosomes 1 (2 clusters), 3 (1 cluster), and 9 (1 cluster) were identified, of which cluster 1 contained three genes (*ZmHSP20-2* to *ZmHSP20-4*), cluster 2 contained three genes (*ZmHSP20-5* to *ZmHSP20-7*), cluster 3 contained five genes (*ZmHSP20-16* to *ZmHSP20-20*), and cluster 4 contained three genes (*ZmHSP20-40* to *ZmHSP20-42*) (Figure 1). Moreover, six pairs of *ZmHSP20s* exhibited collinearity, which included *ZmHSP20-8* and *ZmHSP20-11*, *ZmHSP20-11* and *ZmHSP20-24*, *ZmHSP20-12* and *ZmHSP20-44*, *ZmHSP20-16* and *ZmHSP20-34*, *ZmHSP20-22* and *ZmHSP20-39*, and *ZmHSP20-30* and *ZmHSP20-36*. 

**Figure 2:**
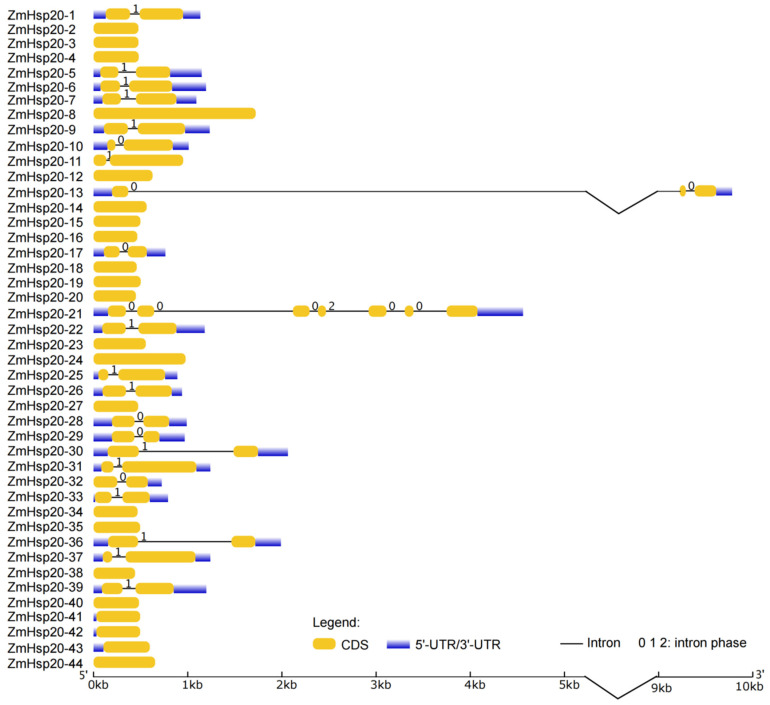
The gene structure of *ZmHsp20s*. The CDSs are displayed with yellow rectangles. The introns are displayed with black lines. Purple rectangles represent UTR. CDS, coding sequence; UTR, untranslated region.

**Figure 3:**
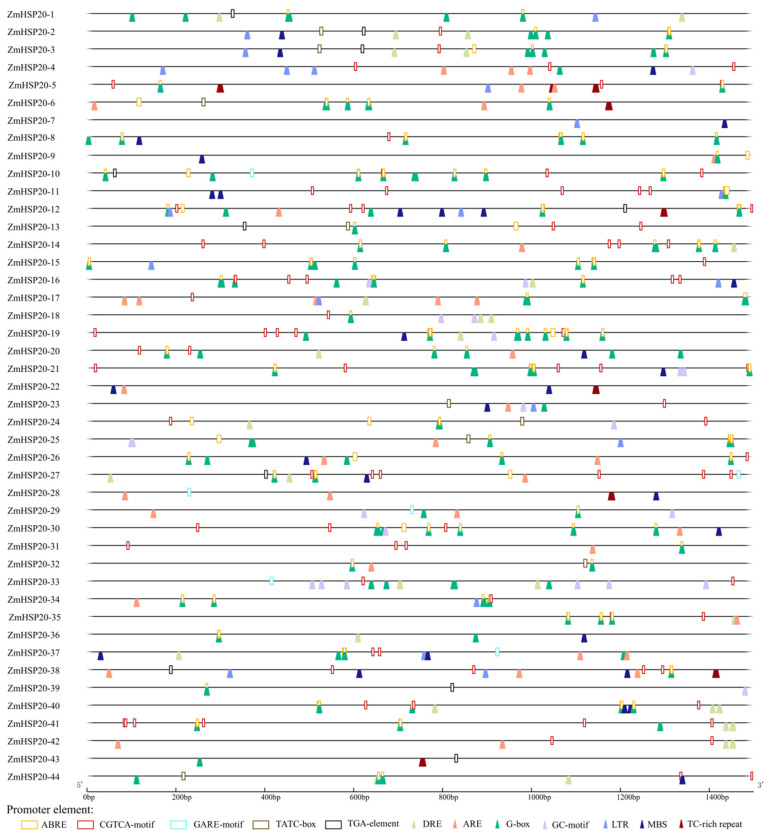
Characters of cis-elements in promoter regions of *ZmHSP20s*. Cis-elements related to hormone responsiveness are represented as cylindrical and cis-elements related to abiotic stress responsiveness are represented as a wedge. ABRE was the response to ABA; ARE and GC-motif were the response to anaerobic conditions; CGTCA-motif was the response to MeJA; DRE and MBS were the response to drought; G-box was light response, GARE-motif and TATC-box were the response to GA; LTR was the response to low temperature; TC-rich repeat was the response to defense and stress; and TGA-element was the response to auxin.

Of 44 *ZmHSP20s*, 22 members had no intron, 20 members had only 1 intron, and 1 member (*ZmHSP20-21*) had 6 introns (Figure 2). One gene, *ZmHSP20-13*, had an ultra-long intron. Seventeen *ZmHSP20s* did not predict the 5′-UTR and 3′-UTR regions. To explore the potential regulatory and function of *ZmHSP20* genes, the cis-acting elements of the *ZmHSP20s* promoter region involved in hormone stimulus and stress response were analyzed (Figure 3). Ten elements involved in hormone stimulus were detected, including the ABA response element (ABRE), auxin response element (TGA-element, AuxRR-core), GA response element (TATC-box, GARE-motif, and P-box), MeJA response element (CGTCA-motif and TGACG-motif), SA response element (TCA-element and SARE). The cis-elements of drought responsiveness (DRE), anaerobic responsiveness (ARE and GC-motif), low-temperature responsiveness (LTR), wound responsiveness (WUN-motif), and light responsiveness (G-box) were also identified. The number of cis-elements ranged from 4 (*ZmHSP20-43*) to 39 (*ZmHSP20-21*). *ZmHSP20-3* had 26 cis-elements, of which 16 cis-elements were related to hormone and abiotic stresses, including ABA, auxin, GA, MeJA, drought, low-temperature, and light. The G-box occupied the most genes, which appeared in the promoter regions of 40 *ZmHSP20* genes, except *ZmHSP20-7*, *ZmHSP20-22*, *ZmHSP20-28*, and *ZmHSP20-42*. ABRE presented in 36 genes, of which *ZmHSP20-19* contained 10, and *ZmHSP20-10* and *ZmHSP20-21* contained 8, respectively. In particular, GC-motif appeared 6 times in *ZmHSP20-33* but no more 2 in the other genes. Moreover, 23 of *ZmHSP20s* contained the MBS element, 10 of *ZmHSP20s* contained the TGA element, and 9 of *ZmHSP20* contained the TATC_box. These results indicated that *ZmHSP20s* were involved in multiple hormonal and abiotic responses.

### 3.2. Conserved Function of ZmHSP20s

**Figure 4:**
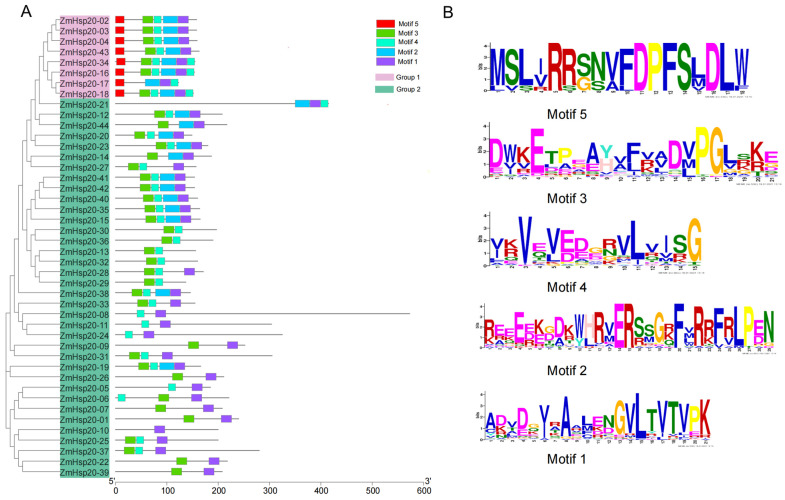
Analysis of the conserved motif in the ZmHSP20s protein. (**A**) Conserved motifs in ZmHSP20 proteins. The phylogenetic tree of ZmHSP20s was constructed with amino acid sequences using MEGA11 software. Different motifs are presented in different colors. ZmHSP20s were classified into group 1 and group 2 based on the presence or absence of Motif 5. (**B**) Motif sequences were predicted in the ZmHSP20s protein. The overall height of the amino acid stacks plotted on the y-axis indicates the sequence conservation at a given position, while the height of individual symbols within a stack indicates the relative frequency of a nucleotide base at that position.

The conserved motifs in ZmHSP20s proteins were analyzed using MEME (Figure 4). A total of five motifs were identified, of which Motif 1 was detected in all ZmHSP20s proteins, and more than two motifs in one protein were identified (Figure 4A). Motif 1, Motif 3, and Motif 4 were distributed on most of the proteins while Motif 5 was only found on 8 members, including ZmHSP20-02, ZmHSP20-03, ZmHSP20-04, ZmHSP20-16, ZmHSP20-17, ZmHSP20-18, ZmHSP20-34, and ZmHSP20-43. These ZmHSP20s were divided into two subgroups based on whether they contained the Motif 5 at the N-terminal. Interestingly, members in group 1 (containing Motif 5) were localized to the cytoplasm and had no intron except ZmHSP20-17 had 1 intron. In particular, ZmHSP20-17 in group 1 lacked Motif 3 compared with other members. The length of these conserved motifs varied from 15 to 29 amino acids (Figure 4B). The GO enrichment analysis of 44 ZmHSP20 genes was conducted, of which 35 genes were enriched (Figure S1). The significant GO terms mainly included the response to hydrogen peroxide, response to hydrogen peroxide, response to reactive oxygen species, response to heat, response to osmotic stress, response to stimulus, and protein oligomerization, indicating the important roles in abiotic stress.

**Figure 5:**
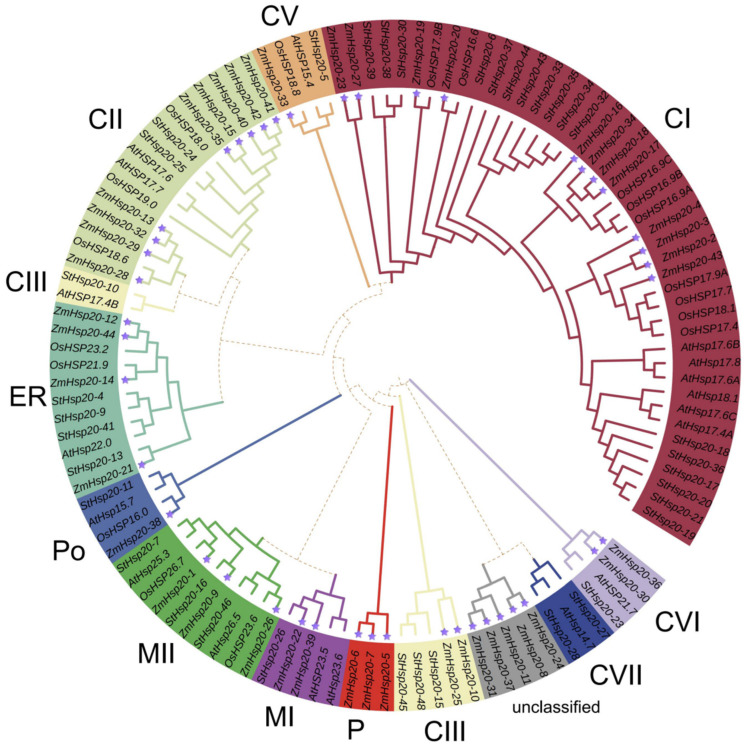
Phylogenetic tree of HSP20 proteins of rice (Os), *Arabidopsis* (At), *Solanum tuberosum* (St)*,* and maize (Zm) using MEGA11 software based on the NJ method. Eleven subfamilies with different colors were classified and unclassified ZmHSP20s are labeled with grey.

To explore the evolutionary relationship of *HSP20s* in plants, 44 of *ZmHSP20s*, 18 of *AtHSP20s*, 18 of *OsHSP20s*, and 35 of *StHSP20s* were subjected to construction of a phylogenetic tree, which was divided into 11 categories according to a previous classification [47,60,61] (Figure 5). These proteins were predicted to localize in 6 organelles, including the cytoplasm and nucleus (C), endoplasmic reticulum (ER), peroxisome (Po), mitochondria (M), and plasma (P). The proteins in the categories of CI, CII, CIII, CV, CVI, and CVII were mainly localized in the cytoplasm and nucleus, proteins in the category of MI and MII were mainly localized in the mitochondria, while proteins in the category of ER, Po, and P were mainly localized in the endoplasmic reticulum, peroxisome, and plasma, respectively. The CI category had the largest number of members, and most of the members in category CII belonged to maize and rice. The category of CV and Po had only four members, with one member of each species. The P category had only three ZmHSP20s (ZmHSP20-5, ZmHSP20-6, and ZmHSP20-7), and the CVII category had three members (AtHSP14.7, StHSP20-27, and StHSP20-28) from dicotyledonous plants, and the CVI category had four members from maize, *Solanum tuberosum*, and *Arabidopsis*. The phylogenetic relationship indicated the conservation and difference in HSP20s in plant evolution. 

### 3.3. High Temperature Strongly Induced the Expression of ZmHSP20s

**Figure 6:**
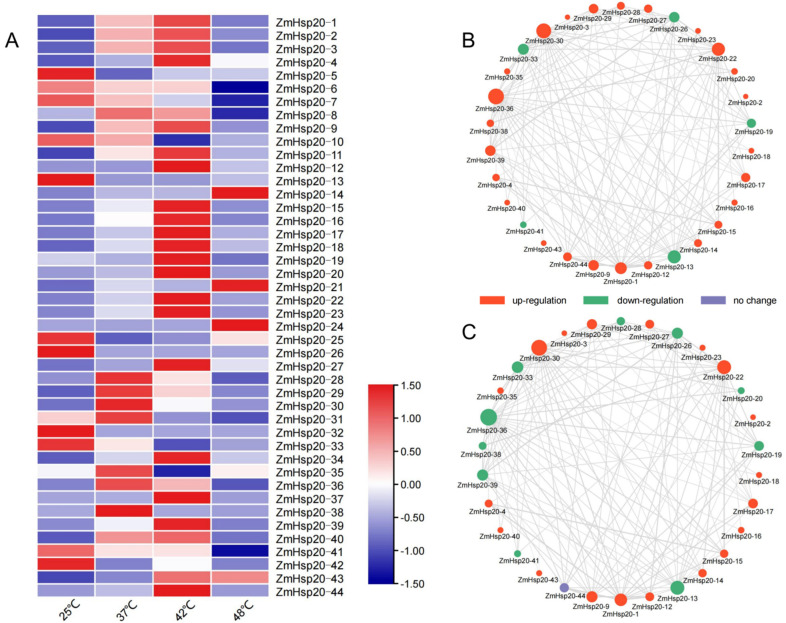
The expression level of *ZmHSP20s* under heat stress. (**A**) Heatmap showing the expression levels of *ZmHSP20s* at 25, 37, 42, and 48 °C based on qRT-PCR. (**B**) The PPI map of ZmHSP20s was drawn and the genes upregulated at 37 °C are shown in red, and the downregulated genes are shown in green. PPI, protein–protein interaction. (**C**) The PPI map of ZmHSP20s was drawn and the genes upregulated at 48 °C are shown in red, and the downregulated genes are shown in green.

To investigate the response of *ZmHSP20s* to high temperature, qRT-PCR was applied to analyze the expression level of 44 *ZmHSP20s* under 37, 42, and 48 °C stresses (Figure 6). Of 44 genes, 31 genes were upregulated after heat stress, while 12 genes such as *ZmHSP20-3*, *ZmHSP20-16*, *ZmHSP20-17*, *ZmHSP20-18*, *ZmHSP20-34*, and *ZmHSP20-43* were increasingly induced under three temperature gradients (Figure 6A). The highest upregulation of *ZmHSP20s* was under 42 °C stress, which was more than 1000-fold compared with the normal condition (25 °C). Only 23 genes were upregulated under 48 °C stress, of which one gene, *ZmHSP20-24*, was only upregulated (116-fold) at this temperature point. One gene, *ZmHSP20-38*, was only upregulated (32-fold) under 37 °C stress. The interaction network of ZmHSP20s showed that only 30 genes interacted with each other (Figure 6B,C). Except for *ZmHSP20-24,* these 14 *ZmHSP20s* that were not in the network were not upregulated by heat stress. We further compared the expression level of *ZmHSP20s* under 37 and 48 °C stresses, and nine genes such as *ZmHSP20-20*, *ZmHSP20-24*, *ZmHSP20-28*, and *ZmHSP20-36 to ZmHSP20-39* displayed opposite expression profiling under 37 and 48 °C stresses (Figure 6B,C). Moreover, a significantly higher expression level of *ZmHSP20s* under 37 and 42 °C stresses than under 48 °C stress was detected (Figure S2), implying the differential expression of *ZmHSP20s* under different degrees of heat stress.

### 3.4. Differential Expression of ZmHSP20s under Hormonal Stimuli and Abiotic Stresses

**Figure 7:**
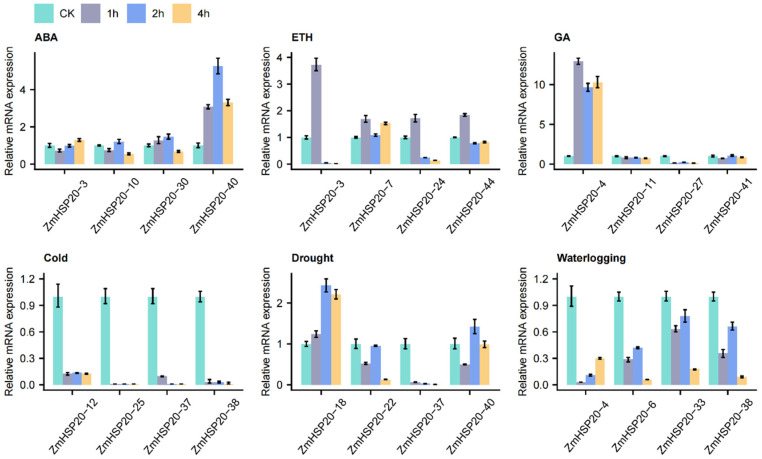
Expression level of *ZmHSP20s* under hormone stimulus and abiotic stresses. The height of each column indicates the mean value of three technical replicates. ABA, abscisic acid; ETH, ethylene; GA, gibberellin; 1, 2, and 4 h indicate the time of treatment.

Given that a large number of cis-elements related to hormone and abiotic response occurred in the promoter region of *ZmHSP20s*, 20 *ZmHSP20s* were randomly selected to analyze their expression level under three treatments of hormone (ABA, ethylene, and GA) and three abiotic stresses (cold, drought, and waterlogging) (Figure 7). Under the ABA treatment, *ZmHSP20-40* was apparently upregulated, while *ZmHSP20-3*, *ZmHSP20-10*, and *ZmHSP20-30* had minor changes. All four genes (*ZmHSP20-3*, *ZmHSP20-7*, *ZmHSP20-24*, and *ZmHSP20-44*) were upregulated under 1 h of ethylene treatment, whereas *ZmHSP20-3* and *ZmHSP20-24* were downregulated under 2 and 4 h of treatment. *ZmHSP20-4* had more than 10-fold induction after GA treatment, while *ZmHSP20-27* was reduced by GA. Cold stress strongly restricted the expression of four genes (*ZmHSP20-12*, *ZmHSP20-25*, *ZmHSP20-37*, and *ZmHSP20-38*), and the expression restriction of *ZmHSP20-4*, *ZmHSP20-6*, *ZmHSP20-33*, and *ZmHSP20-38* was different under waterlogging stress. Furthermore, *ZmHSP20-18* were upregulated after 2 and 4 h of drought stress, whereas *ZmHSP20-37* was strongly limited. These results indicated that the members of *ZmHSP20s* play different roles in different stimuli.

### 3.5. Interaction of the ZmHSP20s with the ZmHSFs

**Figure 8:**
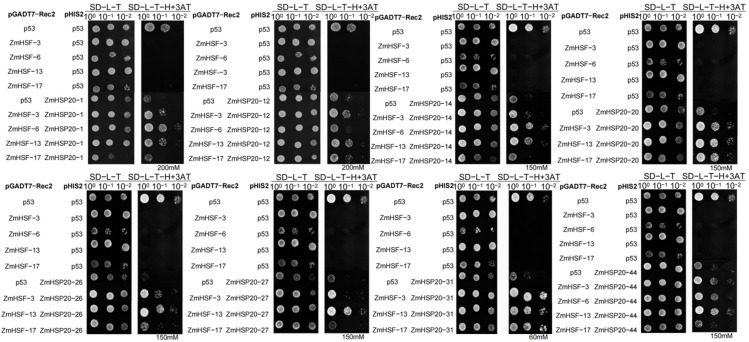
The binding of ZmHSFs with the promoter of *ZmHSP20s* using the yeast-one-hybrid experiment. The p53 represents the positive control; SD, synthetic dropout medium; L, leucine; T, tryptophan; H, histidine; 3-AT, 3-amino-1,2,4-triazole.

The protein interaction between ZmHSP20s and ZmHSFs was predicted using Strings [57] and it was found that 7 of the ZmHSP20s interacted with 14 of the ZmHSFs (Figure S3). To verify their interaction at the protein level, six *ZmHSFs* CDSs (*ZmHSH02*, *ZmHSH10*, *ZmHSH15*, *ZmHSH17*, *ZmHSH24*, and *ZmHSH25*) were inserted into the *pGADT7* vector, and six *ZmHSPs* CDSs (*ZmHSP20-1*, *ZmHSP20-9*, *ZmHSP20-26*, *ZmHSP20-38*, *ZmHSP20-41*, and *ZmHSP20-44*) were inserted into the *pGBKT7* vector (Table S1). Yeast-two-hybrid experiments detected no interaction between ZmHSFs and ZmHSP20s (Figure S4). The predicted binding of ZmHSFs with the promoter sequence of *ZmHSP20s* in PlantRegMap [59] showed that four ZmHSFs (ZmHSF3, ZmHSF6, ZmHSF13, and ZmHSF17) can bind to 32 of the *ZmHSP20s* promoters (Table S3). Yeast-one-hybrid was applied to verify the binding of four ZmHSFs with the promoter sequence of eight *ZmHSP20s* (*ZmHSP20-1*, *ZmHSP20-12*, *ZmHSP20-14*, *ZmHSP20-20*, *ZmHSP20-26*, *ZmHSP20-27*, *ZmHSP20-31*, and *ZmHSP20-44*) (Figure 8). The ZmHSF3 and ZmHSF13 can interact with *ZmHSP20-1*, *ZmHSP20-12*, *ZmHSP20-14*, *ZmHSP20-20*, *ZmHSP20-26*, *ZmHSP20-31*, and *ZmHSP20-44*, ZmHSF6 can interact with *ZmHSP20-1*, *ZmHSP20-14*, *ZmHSP20-20*, and *ZmHSP20-44*, and ZmHSF17 can interact with *ZmHSP20-12*, *ZmHSP20-20*, and *ZmHSP20-31*. The differential strength of interactions between *ZmHSPs* and ZmHSFs was also observed, which included the strong interaction between ZmHSF3, ZmHSF13, and ZmHSF17 with the promoter of *ZmHSP20-20*, *ZmHSP20-26*, and *ZmHSP20-31*.

## 4. Discussion

Abiotic stress hurts crop development and yield and is a major barrier to meeting food demand worldwide. Plants have different strategies for coping with different types of stress. *HSPs* were induced in almost all stresses [2], and each member of the *HSPs* group has a unique roles [62]. *HSP20s* is a subfamily of *HSPs* groups, which is also called small *HSPs*. The expression levels of *HSP20s* were regulated by heat, salt, and powdery mildew in barley (*Hordeum vulgare* L.) [63], and the expression of *Lilium davidii HSP16.45* in *Arabidopsis thaliana* enhanced the latter cell activity in heat, salt, and oxidative stress [64], indicating that *HSP20s* play essential roles in biotic and abiotic stresses. In the present study, a total of 44 *ZmHSP20s* were identified (Table S2), and four clusters in three chromosomes were detected (Figure 1). The gene structure and amino acid sequence were conserved among 44 members (Figure 2 and Figure 4), and six pairs of genes were collinear, of which these characters were also detected in tomatoes and apples [65,66]. The analysis of the phylogenetic relationship in maize, rice, *Arabidopsis*, and potato demonstrated that the specific subcellular localization of each category was presented, indicating the specific function of *HSP20s* in each category. Some evolution-related categories such as P and CVI were also identified, which may play vital roles in maize and dicotyledonous plants, respectively. Moreover, the member of *OsHSP20s* was not detected in the CVI category, implying the possible association with the aquatic environment. 

Gene expression was strongly affected by environmental stimuli, which was regulated through multiple factors such as cis-elements and trans-factors. The protein of trans-factors can bind to the cis-elements in the promoter to activate or inhibit the expression of targets. The cis-elements in the promoter of one gene can reflect its potential expression profiling. The *HSP20s* participate in diverse biotic and abiotic stresses [49], which implied that some cis-elements related to stresses may be located in the promoter of *HSP20s*. Using the online tool PlantCare [53], the cis-elements in the promoter of 44 *ZmHSP20s* were identified (Figure 3), and large amounts of elements associated with hormone and abiotic stress were detected in all genes, indicating that *ZmHSP20s* are also tightly associated with abiotic stresses. To verify these results, qRT-PCR was conducted to analyze the response of hormone and abiotic stresses (Figure 7). All selected genes responded to hormone stimuli (ABA, GA, and ethylene) and abiotic stresses (hypoxia, low temperature, and drought), of which all four genes increased their expression after 1 h of ethylene, suggesting their possible roles in ethylene-mediated signals. The expression of four *ZmHSP20s* were restricted under cold stress, similar to previous transcriptome analysis [67]. Under given conditions, some *ZmHSP20s* were upregulated while some *ZmHSP20s* were inhibited, demonstrating their differential function in response to stresses.

Heat stress seriously affects growth, development, and yield, which frequently occurs with the increasing global climate. The expression of *HSP20s* was activated, and yielded proteins can avoid protein degradation [13,18], which usually play roles in molecular chaperone, retaining suitable conformations [19,20]. The GO analysis of 44 *ZmHSP20s* displayed that these genes are mainly involved in stresses such as high temperature, osmosis, and salt stress. They also involved in protein assembly, folding, and membrane composition (Figure S1), which were also discovered in rice [68], indicating the conserved characters of *HSP20s* in plants under heat stress. Transcriptome analysis of maize seedling leaves revealed that *ZmHSP20* were obviously upregulated under heat stress [67], and qRT-PCR analysis of *ZmHSP20* under 37, 42, and 48 °C stresses showed that approximately 80% of *ZmHSP20* were upregulated (Figure 6), implying the essential roles of *ZmHSP20s* under heat stress. Moreover, the differential expression profiling of *ZmHSP20s* under 37 and 48 °C conditions indicated their diverse roles. Specifically, the genes in cluster 3 such as *ZmHSP20-16*, *ZmHSP20-17*, and *ZmHSP20-20* were significantly upregulated (more than 1000-fold) under heat stress, which would be a potential target for genetic improvement of heat stress. Moreover, the induced expression of *ZmHSP20s* under heat stress depended on the binding of ZmHSFs proteins in their promoter regions (Figure 8), but not on protein–protein interaction between ZmHSFs and ZmHSP20s (Figure S4), suggesting the molecular mechanism of ZmHSP20s in response to heat stress. 

## Supplementary Materials

The following supporting information can be downloaded at: https://www.mdpi.com/article/10.3390/life12091397/s1, Figure S1. Functional analysis of 44 *ZmHSP20s* based on Gene Ontology. Figure S2. Boxplots showing the expression levels of *ZmHSP20s* under heat stress. Expression levels of *ZmHSP20s* increased under 37 °C, 42 °C, and 48 °C conditions, comparing with 25 °C. Figure S3. Predicted Interaction between ZmHSP20s and ZmHSFs protein using STRING database. Figure S4. The interaction between ZmHSFs and ZmHSP20s based on yeast-two-hybrid experiments. SD, synthetic dropout medium; Leu, leucine; Trp, tryptophan; His, histidine; Ade, adenine. Table S1. The primer using in this study. Table S2. Information of 44 ZmHSP20s, including Gene, Gene ID, position start, position end, chromosome, isoelectric point (PI), molecular weight (MW), and predicted subcellular localization. Table S3. Predicted gene fragments for ZmHSP20 and HSF interaction.

## References

[1] Levitt J. (1980). Responses of Plants to Environmental Stresses.

[2] Al-Whaibi M.H. (2011). Plant Heat-Shock Proteins: A Mini Review. J. King Saud Univ. Sci..

[3] Wang W., Vinocur B., Altman A. (2003). Plant Responses to Drought, Salinity and Extreme Temperatures: Towards Genetic Engineering for Stress Tolerance. Planta.

[4] Nakamoto H., Vígh L. (2007). The Small Heat Shock Proteins and Their Clients. Cell. Mol. Life Sci..

[5] Wahid A., Gelani S., Ashraf M., Foolad M. (2007). Heat Tolerance in Plants: An Overview. Environ. Exp. Bot..

[6] Shao H.B., Guo Q.J., Chu L.Y., Zhao X.N., Su Z.L., Hu Y.C., Cheng J.F. (2007). Understanding Molecular Mechanism of Higher Plant Plasticity under Abiotic Stress. Colloids Surf. B.

[7] Mittler R. (2006). Abiotic Stress, the Field Environment and Stress Combination. Trends Plant Sci..

[8] Ritossa F. (1962). A New Puffing Pattern Induced by Temperature Shock and DNP in Drosophila. Experientia.

[9] Lindquist S., Craig E.A. (1988). The Heat-Shock Proteins. Annu. Rev. Genet..

[10] Gupta S.C., Sharma A., Mishra M., Mishra R.K., Chowdhuri D.K. (2010). Heat Shock Proteins in Toxicology: How Close and How Far?. Life Sci..

[11] De Maio A. (1999). Heat Shock Proteins: Facts, Thoughts, and Dreams. Shock.

[12] Helm K.W., LaFayette P.R., Nagao R.T., Key J.L., Vierling E. (1993). Localization of Small Heat Shock Proteins to the Higher Plant Endomembrane System. Mol. Cell. Biol..

[13] Wu J., Gao T., Hu J., Zhao L., Yu C., Ma F. (2022). Research Advances in Function and Regulation Mechanisms of Plant Small Heat Shock Proteins (SHSPs) under Environmental Stresses. Sci. Total Environ..

[14] Pelham H.R.B. (1982). A Regulatory Upstream Promoter Element in the Drosophila Hsp70 Heat-Shock Gene. Cell.

[15] Waters E.R., Vierling E. (2020). Plant Small Heat Shock Proteins—Evolutionary and Functional Diversity. New Phytol..

[16] Waters E.R. (2013). The Evolution, Function, Structure, and Expression of the Plant SHSPs. J. Exp. Bot..

[17] Banerjee A., Roychoudhury A. (2018). Small Heat Shock Proteins. Plant Metabolites and Regulation Under Environmental Stress.

[18] Ferguson D.L., Guikema J.A., Paulsen G.M. (1990). Ubiquitin Pool Modulation and Protein Degradation in Wheat Roots during High Temperature Stress. Plant Physiol..

[19] Miernyk J.A. (1999). Protein Folding in the Plant Cell. Plant Physiol..

[20] Sun W., Van Montagu M., Verbruggen N. (2002). Small Heat Shock Proteins and Stress Tolerance in Plants. Biochim. Biophys. Acta Gene Struct. Expr..

[21] Morimoto R.I. (1993). Cells in Stress: Transcriptional Activation of Heat Shock Genes. Science.

[22] Neta-Sharir I., Isaacson T., Lurie S., Weiss D. (2005). Dual Role for Tomato Heat Shock Protein 21: Protecting Photosystem II from Oxidative Stress and Promoting Color Changes during Fruit Maturation. Plant Cell.

[23] Li J., Zhang J., Jia H., Li Y., Xu X., Wang L., Lu M. (2016). The Populus Trichocarpa PtHSP17.8 Involved in Heat and Salt Stress Tolerances. Plant Cell Rep..

[24] Sun L., Liu Y., Kong X., Zhang D., Pan J., Zhou Y., Wang L., Li D., Yang X. (2012). ZmHSP16.9, a Cytosolic Class I Small Heat Shock Protein in Maize (*Zea mays*), Confers Heat Tolerance in Transgenic Tobacco. Plant Cell Rep..

[25] He Y., Yao Y., Li L., Li Y., Gao J., Fan M. (2021). A Heat-Shock 20 Protein Isolated from Watermelon (ClHSP22.8) Negatively Regulates the Response of Arabidopsis to Salt Stress via Multiple Signaling Pathways. PeerJ.

[26] Zhang N., Zhao H., Shi J., Wu Y., Jiang J. (2020). Functional Characterization of Class I SlHSP17.7 Gene Responsible for Tomato Cold-Stress Tolerance. Plant Sci..

[27] Guo L.M., Li J., He J., Liu H., Zhang H.M. (2020). A Class I Cytosolic HSP20 of Rice Enhances Heat and Salt Tolerance in Different Organisms. Sci. Rep..

[28] Mahuku G., Lockhart B.E., Wanjala B., Jones M.W., Kimunye J.N., Stewart L.R., Cassone B.J., Sevgan S., Nyasani J.O., Kusia E. (2015). Maize Lethal Necrosis (MLN), an Emerging Threat to Maize-Based Food Security in Sub-Saharan Africa. Phytopathology.

[29] Strable J., Scanlon M.J. (2009). Maize (*Zea mays*): A Model Organism for Basic and Applied Research in Plant Biology. Cold Spring Harb. Protoc..

[30] Han Z., Ku L., Zhang Z., Zhang J., Guo S., Liu H., Zhao R., Ren Z., Zhang L., Su H. (2014). QTLs for Seed Vigor-Related Traits Identified in Maize Seeds Germinated under Artificial Aging Conditions. PLoS ONE.

[31] Xing L.-M., Lyu W.Z., Lei W., Liang Y.-H., Lu Y., Chen J.-Y. (2018). Response of HSP20 Genes to Artificial Aging Treatment in Maize Embryo. Zuo Wu Xue Bao.

[32] Yu F., Liang K., Fang T., Zhao H., Han X., Cai M., Qiu F. (2019). A Group VII Ethylene Response Factor Gene, ZmEREB180, Coordinates Waterlogging Tolerance in Maize Seedlings. Plant Biotechnol. J..

[33] Livak K.J., Schmittgen T.D. (2001). Analysis of Relative Gene Expression Data Using Real-Time Quantitative PCR and the 2^−ΔΔCT^ Method. Methods.

[34] Punta M., Coggill P.C., Eberhardt R.Y., Mistry J., Tate J., Boursnell C., Pang N., Forslund K., Ceric G., Clements J. (2012). The Pfam Protein Families Database. Nucleic Acids Res..

[35] Jiao Y., Peluso P., Shi J., Liang T., Stitzer M.C., Wang B., Campbell M.S., Stein J.C., Wei X., Chin C.S. (2017). Improved Maize Reference Genome with Single-Molecule Technologies. Nature.

[36] Eddy S.R. (1996). Hidden Markov Models. Curr. Opin. Struct. Biol..

[37] Chen X., Lin S., Liu Q., Huang J., Zhang W., Lin J., Wang Y., Ke Y., He H. (2014). Expression and Interaction of Small Heat Shock Proteins (SHsps) in Rice in Response to Heat Stress. Biochim. Biophys. Acta Proteins Proteom..

[38] Lamesch P., Berardini T.Z., Li D., Swarbreck D., Wilks C., Sasidharan R., Muller R., Dreher K., Alexander D.L., Garcia-Hernandez M. (2012). The Arabidopsis Information Resource (TAIR): Improved Gene Annotation and New Tools. Nucleic Acids Res..

[39] Kawahara Y., de la Bastide M., Hamilton J.P., Kanamori H., McCombie W.R., Ouyang S., Schwartz D.C., Tanaka T., Wu J., Zhou S. (2013). Improvement of the Oryza Sativa Nipponbare Reference Genome Using next Generation Sequence and Optical Map Data. Rice.

[40] Letunic I., Khedkar S., Bork P. (2021). SMART: Recent Updates, New Developments and Status in 2020. Nucleic Acids Res..

[41] Ison J., Kalas M., Jonassen I., Bolser D., Uludag M., McWilliam H., Malone J., Lopez R., Pettifer S., Rice P. (2013). EDAM: An Ontology of Bioinformatics Operations, Types of Data and Identifiers, Topics and Formats. Bioinformatics.

[42] Small I., Peeters N., Legeai F., Lurin C. (2004). Predotar: A Tool for Rapidly Screening Proteomes ForN-Terminal Targeting Sequences. Proteomics.

[43] Almagro Armenteros J.J., Salvatore M., Emanuelsson O., Winther O., von Heijne G., Elofsson A., Nielsen H. (2019). Detecting Sequence Signals in Targeting Peptides Using Deep Learning. Life Sci. Alliance.

[44] Yu C.S., Lin C.J., Hwang J.K. (2004). Predicting Subcellular Localization of Proteins for Gram-Negative Bacteria by Support Vector Machines Based on n -Peptide Compositions. Protein Sci..

[45] Hu B., Jin J., Guo A.-Y., Zhang H., Luo J., Gao G. (2015). GSDS 2.0: An Upgraded Gene Feature Visualization Server. Bioinformatics.

[46] Wang Y., Tang H., DeBarry J.D., Tan X., Li J., Wang X., Lee T.-H., Jin H., Marler B., Guo H. (2012). MCScanX: A Toolkit for Detection and Evolutionary Analysis of Gene Synteny and Collinearity. Nucleic Acids Res..

[47] Siddique M., Gernhard S., von Koskull-Döring P., Vierling E., Scharf K.-D. (2008). The Plant SHSP Superfamily: Five New Members in Arabidopsis Thaliana with Unexpected Properties. Cell Stress Chaperones.

[48] Ouyang Y., Chen J., Xie W., Wang L., Zhang Q. (2009). Comprehensive Sequence and Expression Profile Analysis of Hsp20 Gene Family in Rice. Plant Mol. Biol..

[49] Zhao P., Wang D., Wang R., Kong N., Zhang C., Yang C., Wu W., Ma H., Chen Q. (2018). Genome-Wide Analysis of the Potato Hsp20 Gene Family: Identification, Genomic Organization and Expression Profiles in Response to Heat Stress. BMC Genom..

[50] Li K.-B. (2003). ClustalW-MPI: ClustalW Analysis Using Distributed and Parallel Computing. Bioinformatics.

[51] Tamura K., Stecher G., Kumar S. (2021). MEGA11: Molecular Evolutionary Genetics Analysis Version 11. Mol. Biol. Evol..

[52] Letunic I., Bork P. (2021). Interactive Tree Of Life (ITOL) v5: An Online Tool for Phylogenetic Tree Display and Annotation. Nucleic Acids Res..

[53] Lescot M. (2002). PlantCARE, a Database of Plant Cis-Acting Regulatory Elements and a Portal to Tools for in Silico Analysis of Promoter Sequences. Nucleic Acids Res..

[54] Chen C., Chen H., Zhang Y., Thomas H.R., Frank M.H., He Y., Xia R. (2020). TBtools: An Integrative Toolkit Developed for Interactive Analyses of Big Biological Data. Mol. Plant.

[55] Zhang H., Li G., Fu C., Duan S., Hu D., Guo X. (2020). Genome-Wide Identification, Transcriptome Analysis and Alternative Splicing Events of Hsf Family Genes in Maize. Sci. Rep..

[56] Woodhouse M.R., Cannon E.K., Portwood J.L., Harper L.C., Gardiner J.M., Schaeffer M.L., Andorf C.M. (2021). A Pan-Genomic Approach to Genome Databases Using Maize as a Model System. BMC Plant Biol..

[57] Szklarczyk D., Gable A.L., Nastou K.C., Lyon D., Kirsch R., Pyysalo S., Doncheva N.T., Legeay M., Fang T., Bork P. (2021). The STRING Database in 2021: Customizable Protein–Protein Networks, and Functional Characterization of User-Uploaded Gene/Measurement Sets. Nucleic Acids Res..

[58] Shannon P., Markiel A., Ozier O., Baliga N.S., Wang J.T., Ramage D., Amin N., Schwikowski B., Ideker T. (2003). Cytoscape: A Software Environment for Integrated Models of Biomolecular Interaction Networks. Genome Res..

[59] Tian F., Yang D.C., Meng Y.Q., Jin J., Gao G. (2019). PlantRegMap: Charting Functional Regulatory Maps in Plants. Nucleic Acids Res..

[60] Vierling E., Harris L.M., Chen Q. (1989). The Major Low-Molecular-Weight Heat Shock Protein in Chloroplasts Shows Antigenic Conservation among Diverse Higher Plant Species. Mol. Cell. Biol..

[61] Sarkar N.K., Kim Y.-K., Grover A. (2009). 099 Rice SHsp Genes: Genomic Organization and Expression Profiling under Stress and Development. BMC Genom..

[62] Panaretou B., Zhai C. (2008). The Heat Shock Proteins: Their Roles as Multi-Component Machines for Protein Folding. Fungal Biol. Rev..

[63] Li J., Liu X. (2019). Genome-Wide Identification and Expression Profile Analysis of the Hsp20 Gene Family in Barley (*Hordeum vulgare* L.). PeerJ.

[64] Mu C., Zhang S., Yu G., Chen N., Li X., Liu H. (2013). Overexpression of Small Heat Shock Protein LimHSP16.45 in Arabidopsis Enhances Tolerance to Abiotic Stresses. PLoS ONE.

[65] Yao F., Song C., Wang H., Song S., Jiao J., Wang M., Zheng X., Bai T. (2020). Genome-Wide Characterization of the HSP20 Gene Family Identifies Potential Members Involved in Temperature Stress Response in Apple. Front. Genet..

[66] Yu J., Cheng Y., Feng K., Ruan M., Ye Q., Wang R., Li Z., Zhou G., Yao Z., Yang Y. (2016). Genome-Wide Identification and Expression Profiling of Tomato Hsp20 Gene Family in Response to Biotic and Abiotic Stresses. Front. Plant Sci..

[67] Li Y., Wang X., Li Y., Zhang Y., Gou Z., Qi X., Zhang J. (2020). Transcriptomic Analysis Revealed the Common and Divergent Responses of Maize Seedling Leaves to Cold and Heat Stresses. Genes.

[68] Sarkar N.K., Kim Y.K., Grover A. (2014). Coexpression Network Analysis Associated with Call of Rice Seedlings for Encountering Heat Stress. Plant Mol. Biol..

