# Supplementary material for: Genome-Wide Identification and Characterization of Heat Shock Protein 20 Genes in Maize
Source: Life (Basel). 2022 Sep 8;12(9):1397. doi: 10.3390/life12091397 (PMC9505046; doi:10.3390/life12091397)
Supplement: Supplementary file 1 [file life-12-01397-s001.zip › life-1901038-Supplementary Figure.pdf]

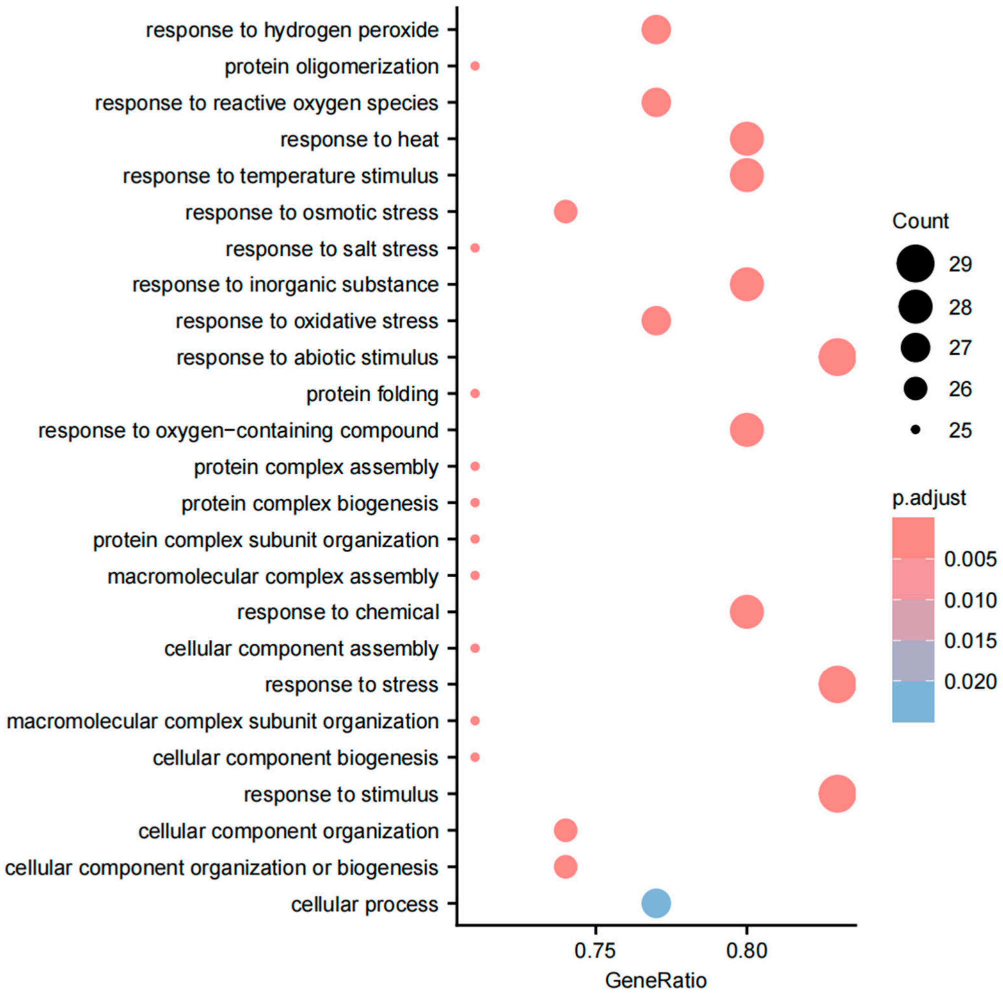

Figure S1. Functional analysis of 44 *ZmHSP20s* based on Gene Ontology.

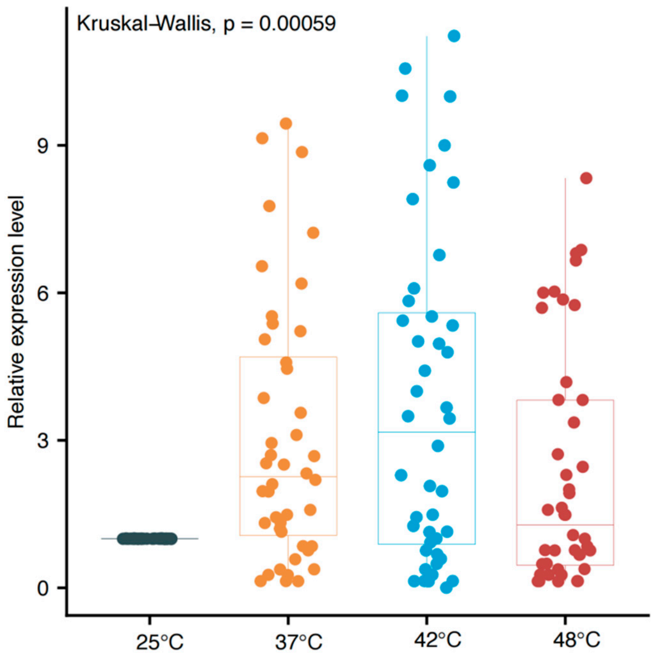

Figure S2. Boxplots showing the expression levels of *ZmHSP20s* under heat stress. Expression levels of *ZmHSP20s* increased under 37 °C, 42 °C, and 48 °C conditions, comparing with 25 °C.

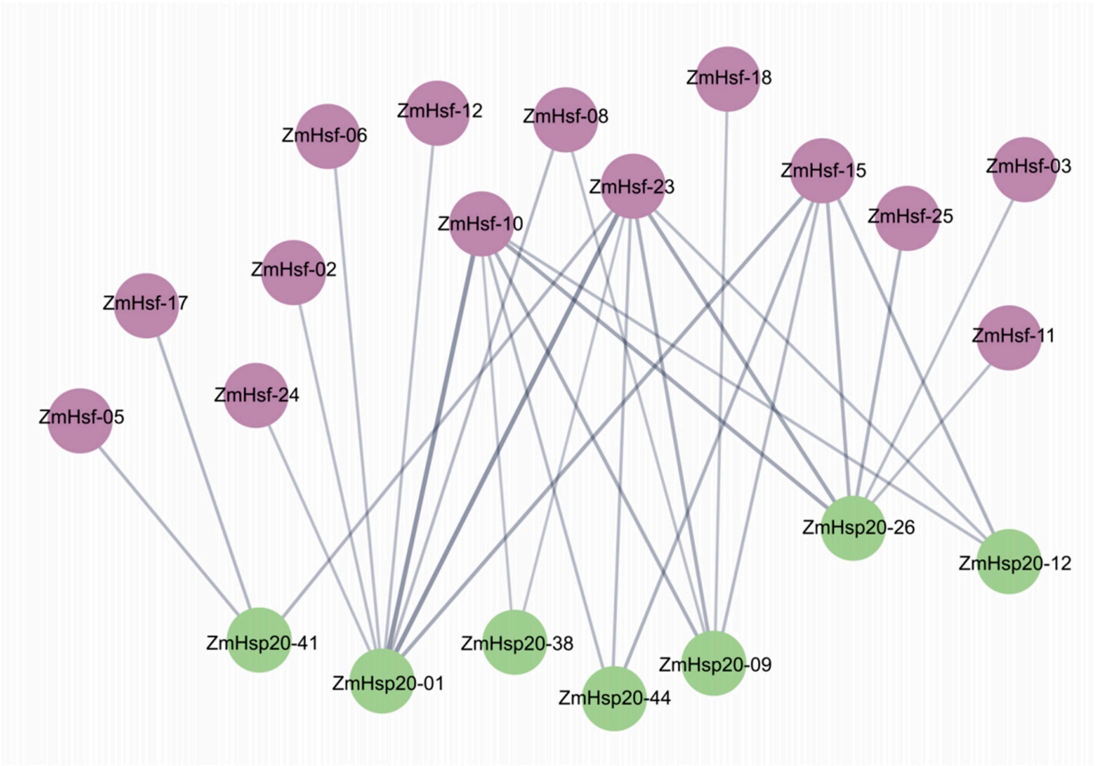

Figure S3. Predicted Interaction between ZmHSP20s and ZmHSFs protein using STRING database.

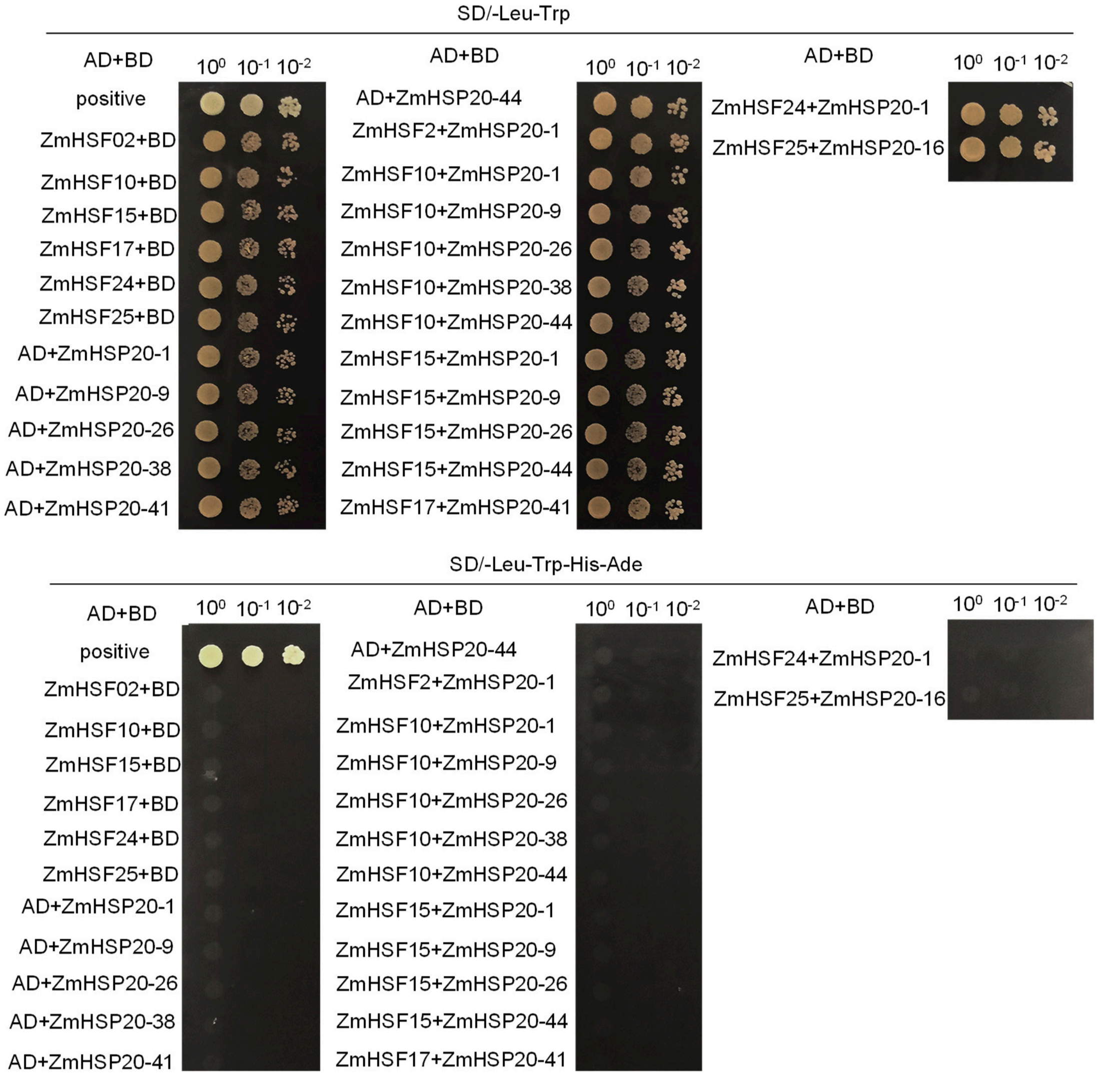

Figure S4. The interaction between ZmHSFs and ZmHSP20s based on yeast-two-hybrid experiments. SD, synthetic dropout medium; Leu, leucine; Trp, tryptophan; His, histidine; Ade, adenine.
